# Controlling a Swarm of Low-Cost Underwater Vehicles Under Conditions of Limited Navigation, Communication and Observation

**DOI:** 10.3390/s26144564

**Published:** 2026-07-18

**Authors:** Tomasz Praczyk, Leszek Pietrukaniec, Maksymilian Wrzesień, Maciej Szymkowiak, Jakub Stalica

**Affiliations:** 1 Computer Science Department, Polish Naval Academy, Smidowicza 69, 81-127 Gdynia, Poland; t.praczyk@amw.gdynia.pl (T.P.);; 2 Naval Weapon Department, Polish Naval Academy, Smidowicza 69, 81-127 Gdynia, Poland

**Keywords:** swarm, underwater vehicles, data-driven model, neural networks

## Abstract

This paper addresses the problem of controlling autonomous underwater vehicles (AUVs) operating in a swarm under realistic underwater conditions characterised by inaccurate navigation, limited acoustic communication, and noisy sonar observations. A novel Trail Sonar-Based Algorithm (TSBA) is proposed for leader–follower swarm control. Unlike conventional reactive approaches, TSBA combines sparse acoustic communication with prior knowledge of the mission plan, enabling predictive estimation of the tracked vehicle’s state and reducing the dependence on continuous information exchange. To evaluate its effectiveness, TSBA was compared with a machine learning-based controller (NSCSUV) in a simulation environment incorporating navigation drift, sonar measurement errors, and a data-driven model of a real low-cost AUV. The proposed vehicle model achieved a mean speed error of 0.107 m/s and a mean heading error of 14.25°, providing a realistic basis for controller evaluation. Simulation results demonstrated that TSBA consistently outperformed the neural network-based approach in formation keeping while generating smoother control commands and requiring only minimal underwater communication. The algorithm maintained stable swarm behaviour despite sensor inaccuracies and communication constraints. Finally, experiments conducted with a real underwater vehicle confirmed the practical applicability and robustness of the proposed approach under real operating conditions.

## 1. Introduction

Autonomous underwater vehicles (AUVs) have become important robotic platforms for performing persistent, repeatable, and risk-reducing operations in underwater environments. Their applications include seabed imaging and mapping, hydrographic and oceanographic surveys, environmental monitoring, inspection of subsea infrastructure such as pipelines, cables and offshore installations, aquaculture monitoring, underwater archaeology, search-and-rescue support, and naval tasks such as mine countermeasures, reconnaissance, and rapid environmental assessment [1,2,3,4]. In many of these missions, the use of autonomous vehicles reduces the need for direct human intervention, limits operational risk for divers, and enables data acquisition in areas where surface communication, satellite navigation, and continuous operator supervision are unavailable or severely constrained [1,3,4].

A natural extension of single-AUV operations is the use of multiple cooperating AUVs organised as teams or swarms. Multi-AUV systems can increase spatial coverage, improve mission efficiency, provide redundancy in case of individual vehicle failure, and distribute sensing or communication tasks among several smaller and less expensive platforms [5,6,7]. Such properties are particularly important in seabed monitoring, infrastructure inspection, search missions, and underwater sensor-network applications, where the mission area may be too large or too uncertain for a single vehicle to cover efficiently [5,8]. However, the practical implementation of cooperative underwater systems remains significantly more difficult than in aerial or ground robotics because underwater vehicles operate under limited navigation accuracy, low-bandwidth and unreliable acoustic communication, and noisy, range-limited observation conditions [5,6,7].

Generally, the idea of underwater swarms is very attractive. The challenge here is its effective implementation, particularly in swarm systems where close cooperation among swarm members is assumed, based on locally operating sensors. The main factor that makes underwater swarm implementation difficult is the underwater environment, which is significantly more challenging than the surface environment. This environment substantially impacts the AUV’s ability to navigate accurately, seriously impedes communication, and limits the range and accuracy of observation sensors, especially in opaque waters such as the Baltic Sea [9].

Many scientific papers on swarms, including underwater swarms, assume ideal communication conditions among swarm members [10,11,12,13,14,15,16,17]. Communication can essentially occur continuously, and there are no limits to the size of transmitted messages. Assuming these conditions, vehicles can exchange their current position, speed, course, and depth, allowing them to adapt their behaviour to that of other vehicles in the swarm. If this data is also sent and received frequently, then a relatively simple controller is sufficient to maintain the swarm’s formation.

Unfortunately, in the underwater environment, this assumption is entirely incorrect. Current underwater communication systems based on sound-wave transmission significantly limit the bandwidth of the acoustic link, allowing only a single transmission of small data packets [18]. Second, the frequency of data exchange is incomparably lower than in radio communication. Equally important is the unreliability of underwater communication, meaning that messages can be lost without reaching the recipient, for example, due to temporally overlapping messages from multiple sources [19,20,21,22,23,24,25,26,27].

Another common assumption is perfect underwater navigation [28,29,30,31,32]. If we combine it with ideal communication, then airborne swarm systems could, in principle, be easily adapted to the underwater environment, something that seems impossible even to a layman. However, if the assumptions about communication are more realistic, but we still rely on ideal navigation, we will have a solution that works at first glance.

The problem in this case is that underwater navigation accuracy deteriorates over time [33]. It relies on navigation sensors that measure speed and heading, but these sensors are unfortunately not perfect [13]; their accuracy depends on the technology they use. However, the more advanced the technology, the higher the price and, in frequent cases, the weight and size of the device.

The solution mentioned above will work, but its quality will degrade as the swarm members’ navigation deteriorates. The vehicles will simply adapt their behaviour based on their own and their neighbours’ positions. This position will be increasingly subject to error, which means that after a long period of staying underwater in the case of advanced and expensive navigation systems, or shortly after the start of the mission in the case of cheap sensors, the swarm formation established at the beginning of the mission will practically cease to exist.

Unrealistic assumptions also often apply to underwater observation systems such as sonars and cameras [34,35,36]. These systems are used for in-swarm navigation to determine the relative location of neighbours (acoustic systems can also be used for the same purpose—they can measure the distance from the signal source—but are outside the scope of the paper). In this case, the detection range is assumed to be close to the device’s maximum range, and the location of the detected object is assumed to be error-free.

In the case of underwater cameras, the decisive factor in determining the detection range is water transparency, which, for example, is high in the Mediterranean Sea but unfortunately low in the Baltic Sea, limiting the cameras’ range to as few as a few metres.

In sonar systems, detection range depends primarily on the object’s size. Unfortunately, underwater vehicles, especially small ones designed to operate in swarms, generate very weak sonar echoes. This echo is even weaker if the vehicles move in parallel within the swarm at the same depth. In such cases, the only visible element is the rear of the vehicle moving in front. Unfortunately, this is only visible at close range, which is well below the sonar’s maximum range.

A separate issue is the accuracy of observation systems in locating a detected object [37,38]. In the case of a camera, the angular coordinates of the object are determined with high accuracy. Determining the distance is a greater challenge, especially when dealing with an unknown object. Operating within a swarm, however, helps determine the distance accurately. With knowledge of the sizes of other vehicles in the swarm, we can accurately estimate the distance. As already mentioned, the problem with cameras, however, is their short range, which practically limits their usefulness exclusively to collision avoidance.

The sonar range, although limited by the poor visibility of other vehicles in the swarm, is greater than the camera range. The problem in this case, in the context of in-swarm navigation, is that the echoes from neighbouring vehicles are not point-like but rather diffuse. Calculating the centre of gravity of such a spot to locate the neighbours typically results in errors related to both relative bearing and distance.

A completely different problem in many scientific studies examining underwater swarms and verifying proposed control algorithms in simulations is the use of models representing virtual, non-existent vehicles [14,15,34,35,39,40,41].

**Figure 1:**
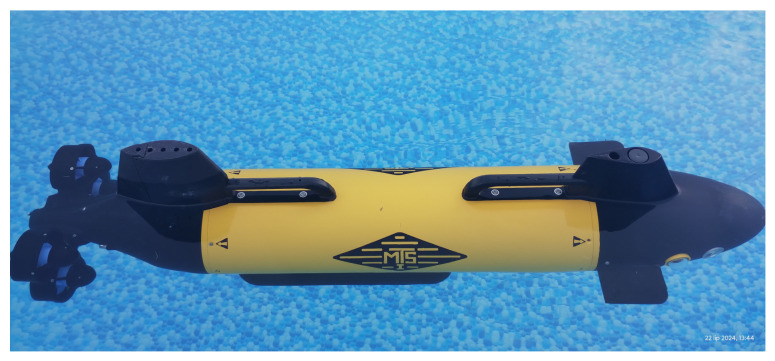
Vehicle used in the tests.

This paper proposes an algorithm for controlling a swarm of underwater vehicles using a leader–follower approach [42,43,44]. This algorithm is a profound modification of the Trail algorithm implemented in the MOOS-IvP framework, and for that reason, it was called the Trail Sonar-Based Algorithm (TSBA). Unlike the original Trail algorithm, TSBA extends it with three key elements: knowledge of the mission plan on the follower’s side, continuous estimation of the tracked object’s state based on that plan, and evolutionarily optimised parametric speed control. These modifications transform the algorithm from a reactive to a predictive one, better adapted to the realities of the underwater environment. To verify the effectiveness of the algorithm, it was compared with the Neural System for Controlling a Swarm of Underwater Vehicles (NSCSUV) algorithm described in [38]. The comparison was conducted under simulation conditions, accounting for all imperfections in underwater navigation, communication, and observation. Furthermore, a data-driven model of a real vehicle (see Figure 1) was used during the simulation, bringing the test conditions closer to real-world conditions. After comparative tests under simulation conditions, a final verification of the proposed algorithm was conducted with real vehicles. The TSBA directly addresses the challenges described above, minimises the use of acoustic communication, mitigates navigation drift through predictive estimation of the neighbour’s state, and accounts for sonar inaccuracy. It should be noted, however, that the TSBA requires a predefined mission plan and exhibits sensitivity to sudden changes in the leader’s speed, which may lead to undesired follower manoeuvres.

The novelty of this work lies in simultaneously addressing three practical limitations that are typically neglected in existing studies on underwater swarms: limited and unreliable acoustic communication, navigation drift due to low-cost sensors, and inaccurate neighbour localisation based on sonar observations. While most existing leader–follower approaches rely on frequent information exchange, accurate navigation systems, or direct measurement of the neighbour’s state, the proposed TSBA utilises prior knowledge of the mission plan to predict the tracked vehicle’s behaviour between successive observations. Consequently, the algorithm significantly reduces the dependence on acoustic communication while maintaining formation under realistic sensing and navigation uncertainties. In addition, unlike studies based on simplified kinematic models, the proposed solution is evaluated using a data-driven model identified from a real underwater vehicle and is subsequently validated in field experiments. To the best of the authors’ knowledge, the combination of predictive mission-plan-based estimation, minimal underwater communication requirements, realistic sensor imperfections, and validation on a real AUV has not been previously reported for leader–follower underwater swarm control.

The contribution of the paper is as follows:

1. A data-driven machine learning (ML)-based underwater vehicle model was proposed and verified.
2. The TSBA algorithm was proposed for follower vehicles tasked with following the leader vehicle in two different formations.
3. The proposed algorithm was compared with an ML-based algorithm under simulation conditions, accounting for all imperfections in underwater navigation, communication, and observation.
4. The final verification of the algorithm’s capabilities was carried out using a real vehicle.

The rest of the paper is organised as follows: Section 2 presents the data-driven vehicle model, which enables realistic simulation of real AUV behaviour. Section 3 describes the TSBA and the NSCSUV used as a reference algorithm. Section 4 presents experiments comparing both algorithms in simulation and verifies the TSBA with real vehicles. The final section summarises the conclusions.

## 2. Vehicle Model

To design an effective control system for a complex tangible object, such as an underwater vehicle shown in Figure 1, tests on this object must always be preceded by tests under simulation conditions. Testing a real object is simply very expensive and time-consuming. However, for tests using a tangible object to be solely a tuning of the system and not a repetition from the very beginning, the object model used in the simulation must be extremely close to the real object. The accuracy of the model is particularly important for vehicles operating in the swarm. When we design a vehicle, say, a classic vehicle that operates for stable values of the desired course, speed, and depth most of the time—i.e., it does not have to manoeuver constantly—then we can be satisfied with an approximate model. However, if our vehicle operates in a swarm of vehicles distant from each other, say, 15–20 m (this is the distance for which a small underwater vehicle is visible using sonar), then the model must be much more accurate.

A natural modelling solution is a traditional model-based approach that employs the laws of physics, fundamentally relying on a set of differential equations that dictate the system’s behaviour. The most prevalent mathematical model for AUV is the six-degree-of-freedom motion model, which varies based on the vehicle’s design [45,46,47,48]. This model has numerous parameters that are easily identifiable, such as weight, geometry, buoyancy, and the number and arrangement of propellers. However, it also requires knowledge of hydrodynamic parameters, such as added mass and linear and nonlinear damping coefficients, making the construction of a complete dynamic model with sufficient accuracy quite challenging, if not unattainable. This is exactly what happened in the case of the model of the testing vehicle. Unfortunately, applying the model-based approach did not yield the expected results, namely a model that reliably approximates vehicle behaviour.

A completely different approach is offered by data-driven black-box methods. In this case, we focus on the observed behaviour of the vehicle in response to control signals. Data-driven models aim to reproduce the behaviour of vehicles they have become familiar with and learned during training. This approach makes such models completely independent of the vehicle design. Knowledge of the internal structure of the vehicle, which is very often difficult to define due to system complexity and environmental variability, is unnecessary. All we need is input–output data that best reflects the behaviour of the vehicle. The most commonly used tool, in this case, is neural networks, ranging from simple perceptron networks (shallow and deep) [49,50,51], convolutional networks (CNNs) [52,53], to recurrent networks (RNNs) [54,55,56], particularly Long Short-Term Memory (LSTM) [57,58,59,60,61] and Gated Recurrent Unit (GRU) networks [62].

The approach outlined above was ultimately applied to the construction of the model for the test vehicle. Unfortunately, in order to obtain a reliable model of the vehicle, a simple approach of using neural networks as a black box that only needs to be fed with training data and whose learning process is carried out was not sufficient. The architecture of the final model resulted from an iterative process in which many different solutions were tested. Ultimately, a modular model was chosen, consisting of two separate models, one for speed and the other for heading. The splitting of the model into two distinct models resulted from the highly unsatisfactory results of the initial attempts to construct a single comprehensive model. Dividing the problem into sub-problems improved the results.

The depth change model was omitted because, according to the assumptions, the swarm the modelled vehicle will be part of will remain at the same depth. This assumption stems from the need to maintain the swarm as a whole, which requires continuous sonar observation of other swarm members. If the vehicles moved at different depths, determining the location of neighbours would be impossible or at least very difficult.

The models specified in this section are adapted to the vehicle design. The most important feature of the vehicle that determines the construction of the models, or rather the input layer of models, is that it has four separate screw propellers: two left and two right. Propellers located on one side of the vehicle operate with the same force. For the vehicle to turn, differential action is required. The vehicle uses the side fins to change depth. However, since the depth change is not modelled, the models presented in this section are not fed with the fin settings.

### 2.1. Speed Model

The speed model (SM) is a modular model with one feed-forward neural network (FFNN) working when the vehicle is assumed to be moving straight ahead and another FFNN when it is assumed to be turning. At each time step *t*, both networks are fed with a sliding window of the input signal LSP(t), RSP(t), consisting of the past *H* observations up to the current time step *t*. Each network has one output and 2H inputs. Formally, the operation of the entire modular model can be defined as follows:

(1) $$V_{M}\left(t\right)=\left\{\begin{array}{cc} V_{max}Out_{S}\left(LSP\left(t\right),RSP\left(t\right),\ldots ,LSP\left(t-H\right),RSP\left(t-H\right)\right) & |\Delta SP\left(t\right)|>SP_{T}\\ V_{max}Out_{T}\left(LSP\left(t\right),RSP\left(t\right),\ldots ,LSP\left(t-H\right),RSP\left(t-H\right)\right) & \mathrm{otherwise} \end{array}\right.$$

where $V_{M}$ is output of the speed model, $V_{max}$ is a maximum vehicle speed measured for maximum force of thrusters, $Out_{S}$, $Out_{T}$ are outputs of “moving straight” and “turning” FFNNs, respectively, LSP(t) and RSP(t) are thrusts on the left and right screw propellers scaled to the range <0,1>, ΔSP(t)=LSP(t)−RSP(t), and $SP_{T}$ is a threshold indicating turn.

### 2.2. Heading Model

The heading model (HM) is based on a recurrent neural network (RNN) that, like the speed model, is supplied with sub-sequences of the length *H*, and it works as follows:

(2) $$\theta_{M}\left(t\right)=\theta_{M}\left(t-1\right)+\Delta \theta_{M}\left(t\right)$$

(3) $$\Delta \theta_{M}\left(t\right)=\left\{\begin{array}{cc} 0 & \Delta SP\left(t\right)\in <SP_{LT}\left(t\right),SP_{RT}\left(t\right)>\\ \theta_{P}Out\left(\left\{LSP{\left(t-\tau \right)}_{\tau =0}^{H}\right\},\left\{RSP{\left(t-\tau \right)}_{\tau =0}^{H}\right\}\right) & \Delta SP\left(t\right)>SP_{RT}\left(t\right)\\ -\theta_{P}Out\left(\left\{LSP{\left(t-\tau \right)}_{\tau =0}^{H}\right\},\left\{RSP{\left(t-\tau \right)}_{\tau =0}^{H}\right\}\right) & \mathrm{otherwise} \end{array}\right.$$

where $\theta_{M}\left(t\right)$ is the output of the heading model, Out is an output of RNN with two inputs, one for LSP and another one for RSP, $\Delta \theta_{M}$ is the change of heading, $\theta_{P}\geq 0$ is a parameter, and $SP_{LT}\left(t\right)$ and $SP_{RT}\left(t\right)$ are thresholds that decide whether the vehicle turns or goes straight ahead. The thresholds depend on the average speed of the vehicle $V_{M,avg}\left(t,H_{avg}\right)$ over $H_{avg}$ last steps: $SP_{LT}\left(t\right)=SP_{L}V_{M,avg}\left(t,H_{avg}\right)$, $SP_{RT}\left(t\right)=SP_{L}V_{M,avg}\left(t,H_{avg}\right)$, where $SP_{L}$ and $SP_{R}$ are parameters.

Although the speed and heading models are separate entities, the same input data that feeds them creates a relationship between vehicle speed and heading, and vice versa. The vehicle turns differently at high and low speeds because its propellers operate differently in each case. A similar relationship exists in the reverse direction. The vehicle changes speed differently when travelling straight and when turning, due to the different propeller operation in each case.

## 3. Control Algorithms

### 3.1. TSBA

The TSBA assumes that each follower tracks one neighbour in the swarm, either the leader or another follower. Furthermore, it is assumed that each follower knows the entire mission plan as a list of waypoints, as well as the number of the current waypoint the leader is moving to. Waypoint parameters are defined as position ${\left(x,y\right)}_{w}$, depth $d_{w}$, speed $V_{w}$, heading $\theta_{w}$, and formation $f_{w}$ on the way to the waypoint. The mission plan is transmitted to the vehicles at the beginning of the mission, when all the vehicles are still on the surface and radio communication is possible, while the current waypoint number is transmitted via an acoustic channel by the leader to all followers and this is the only message sent underwater, limiting the use of the unreliable and “narrow” acoustic channel to a minimum.

An alternative option to the above, which makes more use of the acoustic channel, is for the leader to transmit all parameters of the current waypoint. The frequency of underwater communication in this case is the same as in the previous solution—it is activated only when the waypoint changes. The only difference is an increase in the amount of information transmitted.

The TSBA operates in three stages. First, the state of the tracked object—position, speed, and heading—is estimated (The depth is not estimated because its desired value has been previously sent, the current value is measured with high accuracy, and, furthermore, it is maintained by the depth controllers with great ease.) Next, the track point (indicating the desired follower location, which depends on the swarm’s formation) is determined. Finally, the follower’s speed and heading are determined.

The state estimation of the tracked object is based on information from the follower’s observation system (sonar/camera), enabling determination of the object’s position ${\left(x,y\right)}_{E}\left(t\right)$ in the follower’s coordinate system. In the presence of multiple sonar reflections, the tracked object is selected using a simple heuristic procedure. Initially, GPS data are used to define a region of interest (ROI) and an intensity threshold. Within this ROI, candidate detections are identified as contiguous regions exceeding the average acoustic intensity. In subsequent iterations, the search is limited to the previously defined ROI and expanded if no valid detection is found. If multiple candidates are present, the one closest to the expected object location is selected. This approach may lead to incorrect selection in the presence of objects with similar acoustic characteristics. Additionally, the estimation also assumes that the tracked object is heading to the next waypoint. This allows for heading $\theta_{E}\left(t\right)$ estimation. The estimated object speed $V_{E}\left(t\right)$ corresponds to the speed $V_{w}$ imposed by the mission plan. Unlike position and heading, a single object position observed by sonar/cameras does not allow for a more precise estimation. This requires at least two consecutive positions. However, the imprecision of the estimated positions prevents the system from using them for reliable speed estimation. Furthermore, vehicles in the swarm, attempting to maintain formation, may often accelerate or decelerate between successive sonar scans, complicating accurate speed estimation. Consequently, it is assumed that the tracked object moves at the leader’s speed, i.e., $V_{w}$, on average throughout the mission. Formally, the first stage is defined as follows:

(4) $$V_{E}\left(t\right)=V_{w}$$

(5) $$\theta_{E}\left(t\right)=\left\{\begin{array}{cc} H_{\theta }\left({\left(x,y\right)}_{E}\left(t\right),{\left(x,y\right)}_{w}\right) & t-t_{S}<T_{\theta }\\ H_{\theta }\left({\left(x,y\right)}_{w}^{-1},{\left(x,y\right)}_{w}\right) & \mathrm{otherwise} \end{array}\right.$$

(6) $${\left(x,y\right)}_{E}\left(t\right)=\left\{\begin{array}{cc} P(A_{360}\left(B_{S}\left(t\right)+\theta_{A}\left(t\right)\right),D_{S}\left(t\right),{\left(x,y\right)}_{A}\left(t\right)) & t=t_{S}\\ P(\theta_{E}\left(t\right),V_{E}\left(t\right)\left(t-t_{S}\right),{\left(x,y\right)}_{E}\left(t-\Delta t_{E}\right)) & \mathrm{otherwise} \end{array}\right.$$

where $V_{E}$, $\theta_{E}$, and ${\left(x,y\right)}_{E}$ are the estimated speed, heading, and position of the tracked object, respectively; $H_{\theta }\left(a,b\right)$ calculates heading from point *a* to pont *b*; $t_{S}$ is point in time when sonar/camera provided information to the system; $T_{\theta }$ is a threshold; ${\left(x,y\right)}_{w}^{-1}$ is position of the previous waypoint; P(θ,D,(x,y)) moves point (x,y) in the direction θ by the distance *D*; $A_{360}$ converts input angle to the range <0.360); $B_{S}$ and $D_{S}$ are bearing and distance to the tracked object measured by the sonar/camera; ${\left(x,y\right)}_{A}$ and $\theta_{A}$ are position and heading of the follower estimated by navigation system; and $\Delta t_{E}$ is time interval between successive estimations.

The track point ${\left(x,y\right)}_{T}$ in the second stage is calculated as follows:

(7) $$x_{T}\left(t\right)=x_{E}\left(t\right)+R_{D}cos\left(A_{360}\left(\theta_{E}\left(t\right)+B_{D}\right)\right)$$

(8) $$y_{T}\left(t\right)=y_{E}\left(t\right)+R_{D}sin\left(A_{360}\left(\theta_{E}\left(t\right)+B_{D}\right)\right)$$

where $R_{D}$ and $B_{D}$ are the desired distance and bearing to the tracked object, which are determined based on formation $f_{w}$.

Finally, the desired heading $\theta_{D}$ and speed $V_{D}$ is calculated as follows:

(9) $$\theta_{D}\left(t\right)=H_{\theta }\left({\left(x,y\right)}_{A}\left(t\right),{\left(x,y\right)}_{T}\left(t\right)\right)$$

(10) $$V_{D}\left(t\right)=\left\{\begin{array}{cc} min(p_{1}\left(p_{2}+p_{3}\left(d_{E}^{AT}\left(t\right)-T_{D}^{1}\right)V_{E}\left(t\right),V_{max}^{F}\right) & d_{E}^{AT}\left(t\right)>T_{D}^{1}\\ V_{E}\left(t\right) & T_{D}^{2}<d_{E}^{AT}\left(t\right)\leq T_{D}^{1}\\ p_{4}V_{max}^{F} & \mathrm{otherwise} \end{array}\right.$$

where $V_{max}^{F}$ is the maximum speed of the vehicle, p1..p4 are TSBA parameters, $d_{E}^{AT}$ is the distance between ${\left(x,y\right)}_{A}$ and ${\left(x,y\right)}_{T}$, and $T_{D}^{1}$ and $T_{D}^{2}$ are distance thresholds.

The TSBA algorithm exhibits two significant limitations. First, it assumes that each follower vehicle knows at least the next waypoint assigned to the leader, including the desired speed, depth, heading, and formation parameters. This information enables followers to estimate the leader’s motion parameters, particularly its speed and heading, and consequently maintain the desired formation. However, this assumption is valid primarily when the leader follows relatively long and stable trajectory segments. In scenarios requiring frequent waypoint updates, such as collision-avoidance manoeuvres, the leader would need to repeatedly transmit updated mission parameters via the acoustic communication channel. Due to the limited bandwidth and high latency of underwater acoustic communications, such updates cannot be delivered frequently enough to accurately reflect the leader’s motion. As a result, estimation errors accumulate, degrading formation-keeping performance and potentially compromising the correctness of the swarm configuration. Therefore, TSBA is most effective in missions where the leader follows a relatively predictable trajectory and performs only infrequent manoeuvres.

Second, TSBA employs a simple procedure to determine the desired follower speed and does not incorporate a dedicated speed controller that adapts the commanded velocity based on the distance from the desired formation position. While this approach reduces computational complexity, it may lead to tracking inaccuracies and increased position errors under extreme operating conditions, particularly when large deviations from the desired formation geometry occur.

### 3.2. NSCSUV

NSCSUV is essentially two recurrent neural networks (RNNs) tasked with controlling follower vehicles. Each follower has its own instances of the same networks. The first network, called the Line Formation Neural Network, is launched at the very beginning of the swarm mission, when the vehicles are loosely dispersed near the leader, and is responsible for creating a longitudinal formation in which the vehicles move one behind the other, with the leader at the front. This network is fed with distances from several forward-looking sonar sectors and from the sectors covered by the vehicle’s side cameras.

The second RNN, called the Formation Control Neural Network, is launched after the longitudinal formation is formed and is responsible for maintaining two types of formations by the swarm—one longitudinal and the other wide—in which each follower has a single neighbour to follow. In the longitudinal formation, the neighbour is in front, while in the wide formation, it is to the side. The first formation is used for obstacle avoidance, while the second formation is useful when the swarm searches the seafloor. In this case, the network is fed with the distance $D_{S}$ and bearing $B_{S}$ to the tracked neighbour, along with the desired formation $f_{w}$. $D_{S}$ and $B_{S}$ are measured by sonar while $f_{w}$ is transmitted by the leader via acoustic channel. The NSCSUV, unlike the TSBA, lacks knowledge of the mission plan and, consequently, cannot estimate the expected behaviour of the tracked object in accordance with this plan.

The output layer of both networks is the same and consists of two neurons, the first of which is used to control the speed and the second to control the heading:

(11) $$\theta_{D}\left(t\right)=A_{360}\left(\theta_{A}\left(t\right)+180\Delta t_{D}O_{\theta }\left(t\right)\right)$$

(12) $$V_{D}\left(t\right)=V_{max}^{F}O_{V}\left(t\right)$$

where $\Delta t_{D}$ is the interval between successive network decisions, and $O_{\theta }$, $O_{V}$ are outputs of the network corresponding to heading and speed, respectively.

## 4. Experiments

The entire research was divided into three stages. In the first stage, test vehicle models were constructed, which were necessary for the second simulation stage, in which the TSBA was compared with the NSCSUV. In the final stage, the effectiveness of the TSBA was verified using a testing vehicle.

### 4.1. Designing Vehicle Models

To design the models specified in Section 2, data extracted from the testing vehicle’s logs were used. The dataset was collected during eighteen independent missions performed by the vehicle at a depth of 2 m. During each mission, the vehicle followed a predefined trajectory using onboard PID controllers responsible for heading, speed, and depth control. The controller parameters were determined manually through trial and error. The trajectories consisted of straight sections and turns, providing a representative set of manoeuvres required for model identification.

The vehicle state was recorded using navigation sensors and propulsion system measurements. Navigation parameters were logged at a frequency of 10 Hz, while propulsion commands were recorded at 20 Hz. The shallow operating depth enabled precise GNSS positioning using a buoy equipped with a GNSS antenna attached to the vehicle. These GNSS measurements were integrated within a Kalman filter-based navigation system, providing accurate estimates of vehicle position, speed, and heading. The resulting dataset therefore contained synchronised records of propulsion commands and the corresponding vehicle motion parameters required for learning the relationship between control inputs and vehicle behaviour.

To ensure that the collected data represented a broad range of operating conditions, the missions differed with respect to both trajectory shape and commanded speed:

- 2 rectangle trajectories with left turns and a speed of 1 m/s;
- 2 rectangle trajectories with left turns and a speed of 1.5 m/s;
- 2 rectangle trajectories with left turns and a speed of 2 m/s;
- 2 rectangle trajectories with right turns and a speed of 1 m/s;
- 2 rectangle trajectories with right turns and a speed of 1.5 m/s;
- 2 rectangle trajectories with right turns and a speed of 2 m/s;
- 2 back-and-forth trajectories with a speed of 1 m/s;
- 2 back-and-forth trajectories with a speed of 1.5 m/s;
- 2 back-and-forth trajectories with a speed of 2 m/s.

The eighteen missions formed nine pairs corresponding to identical trajectory types and speed settings. Within each pair, one mission was assigned to the training dataset and the other to the validation dataset, resulting in nine training missions and nine validation missions. This division was performed at the mission level rather than at the sample level in order to prevent information leakage between datasets and to ensure that model performance was evaluated on complete trajectories not used during model optimisation. Consequently, both datasets contained examples of all trajectory classes and speed regimes while remaining mutually independent.

Model optimisation was performed exclusively using the training dataset, whereas the validation dataset was used only after the optimisation process had been completed to assess the generalisation capability of the final models.

Both models were trained using an evolutionary algorithm called Hill Climb Modular Assembler Encoding (HCMAE) [63]. In both cases, not only the weights of the neural networks but also additional model parameters such as $SP_{T}$, $SP_{L}$, $SP_{R}$, $\theta_{P}$ were subject to evolution. Regarding the *H* parameter, which appears in both models, it did not evolve. Each model was optimised for different variants resulting from the used *H*. The following variants were tested: *H* = 3, 5, 10, and 50.

In the case of the speed model, the following fitness function was used:

(13) $$F\left(SM\right)=\frac{1}{1+\sum_{k=1}^{9}\sum_{t=1}^{T_{F}\left(k\right)}\left|V_{T}\left(t,k\right)-V_{M}\left(t,k\right)\right|}$$

where $V_{T}\left(t,k\right)$ is a true speed value read from *t*-th row in *k*-th training log, and $T_{F}\left(k\right)$ is the length of *k*-th training log.

**Figure 2:**
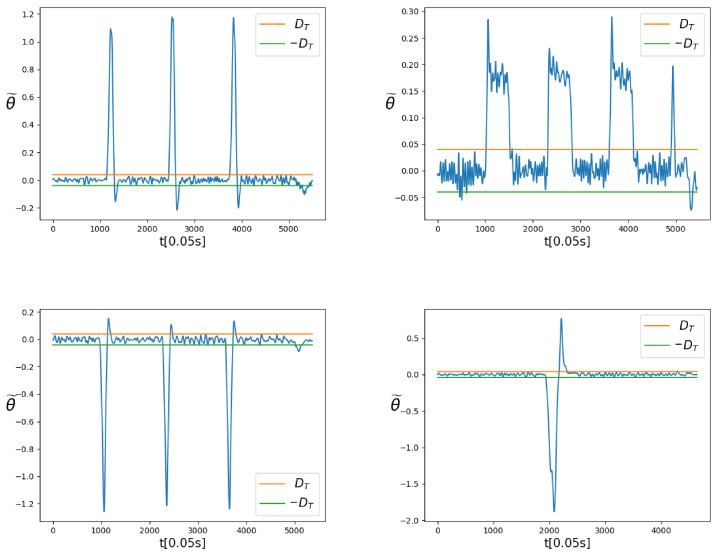
${\tilde{\theta }}^{\prime }$ and $D_{T}$ for four example trajectories.

In the case of heading models, two fitness functions were applied:

(14) $$F_{1}\left(HM\right)=\frac{1}{1+E_{S}+E_{T}}$$

where $E_{S}$, $E_{T}$ are total errors on straight sections of trajectories and turns, respectively:

(15) $$E_{S}=\frac{\sum_{k=1}^{9}\sum_{t=1}^{T_{F}\left(k\right)}\delta \left(t,k\right)\left|\Delta \theta_{M}\left(t,k\right)\right|}{\sum_{k=1}^{9}\sum_{t=1}^{T_{F}\left(k\right)}\delta \left(t,k\right)}$$

(16) $$E_{T}=\frac{\sum_{k=1}^{9}\sum_{t=1}^{T_{F}\left(k\right)}\left(1-\delta \left(t,k\right)\right)\left|d_{ang}\left(\Delta \theta_{T}\left(t,k\right),\Delta \theta_{M}\left(t,k\right)\right)\right|}{\sum_{k=1}^{9}\sum_{t=1}^{T_{F}\left(k\right)}\left(1-\delta \left(t,k\right)\right)}$$

(17) $$\delta \left(t,k\right)=\left\{\begin{array}{cc} 1 & |{\tilde{\theta }}_{k}^{\prime }\left(t\right)|<D_{T}\\ 0 & \mathrm{otherwise} \end{array}\right.$$

where $\Delta \theta_{T}\left(t,k\right)$ is a true change of heading read from logs, $d_{ang}$ is a function calculating the difference between two angles, ${\tilde{\theta }}_{k}^{\prime }\left(t\right)$ is a derivate of continuous heading function generated for *k*-th log at point *t*, and $D_{T}=0.045$ is a threshold that indicates when the vehicle goes straight and when it turns. $D_{T}$ was determined manually after analysis of ${\tilde{\theta }}^{\prime }$ for all logs. Example ${\tilde{\theta }}^{\prime }$ and $D_{T}$ are depicted in Figure 2.

(18) $$F_{2}\left(HM\right)=F_{1}\left(HM\right)+\sum_{k=1}^{9}\sum_{l=1}^{W_{P}}R_{P}\left(l,k\right)$$

where $R_{P}$ is an additional reward for reaching $W_{P}=7$ reference points along each trajectory. The reference points are placed on each straight section of each trajectory before and after the turn. $R_{P}$ is defined as follows:

(19) $$R_{P}\left(l,k\right)=\left\{\begin{array}{cc} 1 & |d_{ang}\left(\theta_{T}\left(\gamma \left(l,k\right),k\right),\theta_{M}\left(\gamma \left(l,k\right),k\right)\right)|\;<T_{H}\left(l\right)\wedge \\ \; & d_{xy}\left(P_{T}\left(l,k\right),P_{M}\left(l,k\right)\right)<T_{xy}\left(l\right)\\ 0 & \mathrm{otherwise} \end{array}\right.$$

where γ(l,k) denotes the row index in the *k*-th log (trajectory) at which the parameters of the *l*-th reference point are stored. Moreover, $d_{xy}$ denotes the Euclidean distance. The point $P_{T}\left(l,k\right)=<x_{T}\left(\gamma \left(l,k\right),k\right),y_{T}\left(\gamma \left(l,k\right),k\right)>$ represents the *l*-th reference point in the *k*-th trajectory, whereas $P_{M}\left(l,k\right)=<x_{M}\left(\gamma \left(l,k\right),k\right),y_{M}\left(\gamma \left(l,k\right),k\right)>$ denotes the model-generated point corresponding to that reference point. Furthermore, $T_{H}\left(l\right)$ and $T_{xy}\left(l\right)$ denote the heading and XY-position thresholds, respectively, used to determine whether the *l*-th reference point has been reached. The threshold values depend on the distance of a given point from the starting point of the trajectory. Specifically, the thresholds are more restrictive for the initial part of the trajectory and less restrictive for later points: $T_{H}\left(l\right)=\left\{2,5,5,10,10,15,15\right\}$ and $T_{xy}\left(l\right)=\left\{5,8,8,15,15,25,25\right\}$.

The results of the best evolved models are presented in Table 1. It contains the maximum errors $E_{max}$, mean errors $E_{mean}$, and standard deviations σ. To design the models, each was evolved 10 times. Once the evolutionary process was completed, each model was tested on the validation trajectories. The models with the smallest maximum error were selected as the final models. The optimised parameters of the final models are as follows: $SP_{T}=0.06$, $SP_{L}=-0.025$, $SP_{R}=0.462$, $\theta_{P}=3.583$. Both final models used the same value of parameter *H*, i.e., H=5.

**Figure 3:**
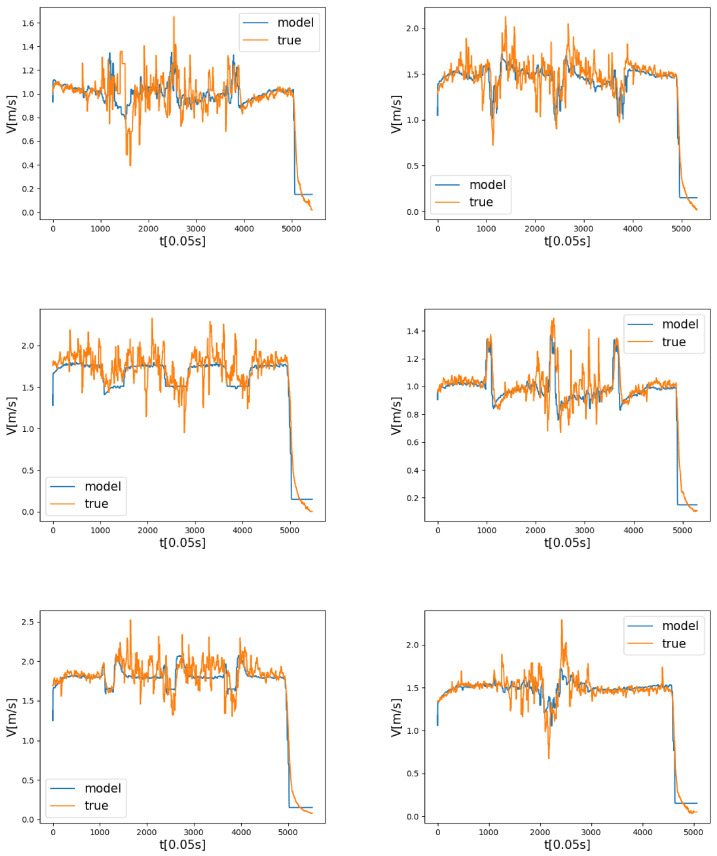
Example results of SM.

**Figure 4:**
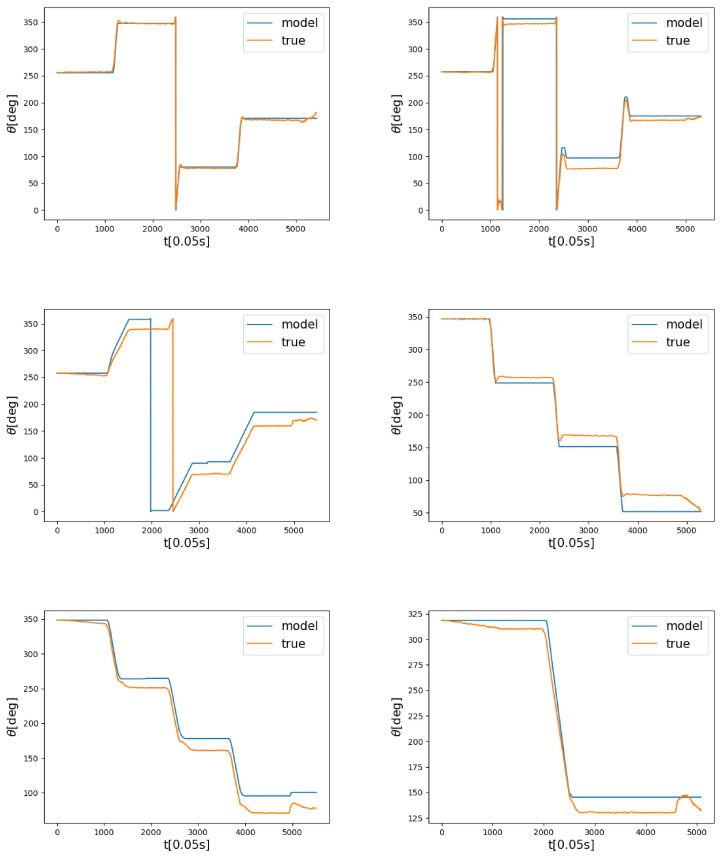
Example results of HM.

**Figure 5:**
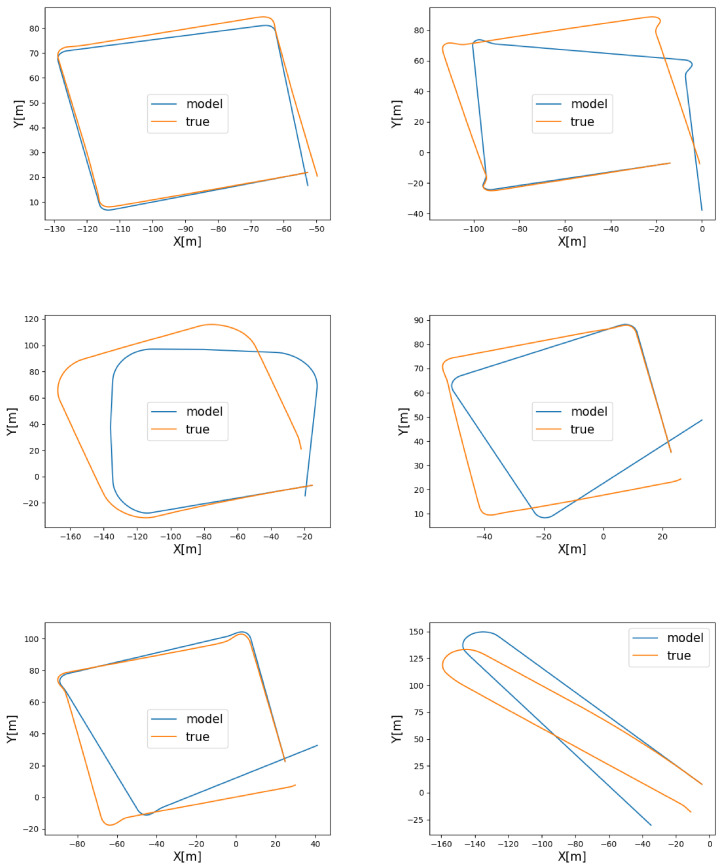
Comparison of true and model trajectories.

**Figure 6:**
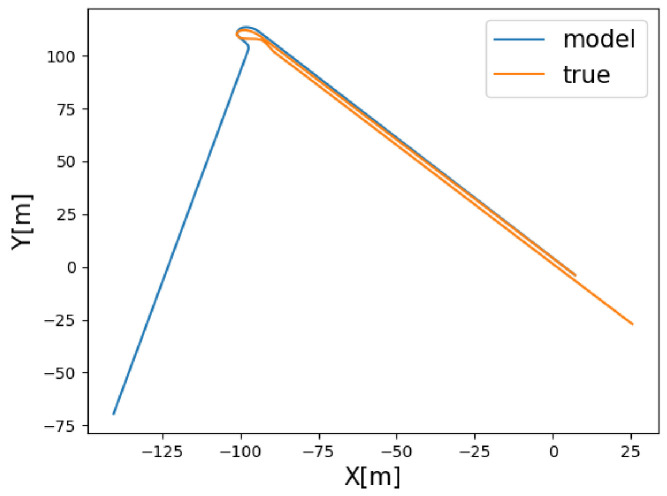
The worst case in heading modelling by HM-RNN2.

The performance of the models on the validation data is presented in Figure 3 and Figure 4, while Figure 5 shows example trajectories generated using both models. The results indicate that the developed models reproduce the vehicle dynamics with sufficient accuracy for control design and simulation studies. The largest HM errors occur during tight turns at higher speeds, particularly when identifying the exact moment at which a turn is completed—see Figure 6. However, these errors are isolated and do not significantly affect the intended application of the model.

The satisfactory modelling accuracy was achieved by separating the model into speed and heading components, modelling straight-line and turning motion separately, and using a fitness function that accounts for these two motion regimes. Although inaccuracies in heading prediction may introduce errors in the predicted position of the tracked vehicle, their influence on TSBA performance is limited because the algorithm does not rely on long-term open-loop prediction. The estimated position is regularly corrected using observation data, while mission-plan information provides additional information about the expected motion of the tracked vehicle. Consequently, model prediction errors do not accumulate over extended periods.

The results presented in Section 4.2 confirm that the achieved model accuracy is sufficient for the considered control application. Despite the remaining modelling inaccuracies, the TSBA maintains stable formation-control performance and generates smooth control signals. Therefore, the developed vehicle model provides an adequate representation of the vehicle dynamics for the design, optimisation, and simulation-based evaluation of the proposed control algorithms.

### 4.2. Comparison Between TSBA and NSCSUV

To compare the algorithms, they were first evolutionarily trained using HCMAC for the swarm consisting of four followers and the leader. The leader’s task was to follow a predefined trajectory without caring for the followers, while the followers’ task was to follow the leader in two formations: narrow and wide. In the narrow formation, the task of the followers was to keep bearing 90 degrees to a neighbour in front, whether it be the leader or a follower, and to maintain a distance of 15 m from it. This distance was experimentally verified as the nearly maximum range of the sonar at which it can detect small vehicles, such as the testing vehicle. In turn, in the wide formation, the followers are tasked with keeping the neighbour centrally in front and maintaining a distance of 5 m from it. The distance of 5 m is small enough to ensure that followers will not confuse the neighbour’s sonar echo with the echo of other objects, e.g., the bottom, which are assumed to be located at a distance greater than 5 m.

The algorithms were trained in an obstacle-free environment, assuming that the only vehicle in the swarm responsible for obstacle detection and collision avoidance is the leader. Upon detecting an obstacle, it orders an avoidance manoeuvre by changing the desired depth for the entire swarm or changing the formation from wide to narrow. The followers use sonar exclusively to track their neighbours, and it is assumed that any echoes beyond 5 m are targets to be ignored.

**Figure 7:**
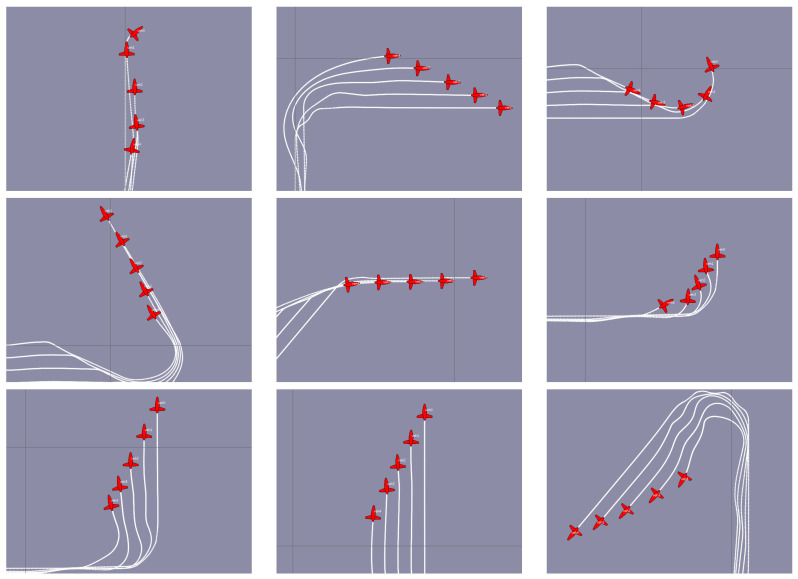
Example of swarm behaviour during training (the alogview Mission Oriented Operating Suite—Interval Programming MOOS-IvP application was used to visualise behaviour).

During training, the swarm’s task was to follow two training trajectories approximately 4000 m long, each consisting of straight sections of varying lengths and left- and right-hand turns. On the turns, the swarm maintained a narrow formation, while on the straight sections, the formation could be either wide or narrow. The swarm’s speed depended on the formation. For a narrow formation, it was always 1 m/s, while for a wide formation, it was either 0.5 m/s or 1 m/s. An example of the swarm’s behaviour along training trajectories is depicted in Figure 7.

The vehicles were modelled using the optimised model specified in Section 2 and equipped with heading and speed PID controllers manually tuned on the testing vehicle. To calculate fitness, a slightly modified variant of the function applied in [38] was used:

(20) $$F_{3}\left(TSBA/NSCSUV\right)=N^{I}+W_{1}\left(B^{narrow}+B^{wide}\right)+W_{2}\left(D^{narrow}+D^{wide}\right)$$

where $N^{I}$ determines the duration of swarm’s mission, $W_{1}$ and $W_{2}$ are weighting parameters, and $B^{narrow}$, $B^{wide}$, $D^{narrow}$, and $D^{wide}$ are defined as follows:

(21) $$B^{narrow}=\sum_{k}^{N^{T}}\sum_{i}^{N^{F}}\sum_{j}^{N^{narrow}}\left(1-\frac{|B_{S,k,i,j}|}{B_{S,max}}\right)$$

(22) $$B^{wide}=\sum_{k}^{N^{T}}\sum_{i}^{N^{F}}\sum_{j}^{N^{wide}}\frac{|B_{S,k,i,j}|}{B_{S,max}}$$

(23) $$D^{narrow}=\sum_{k}^{N^{T}}\sum_{i}^{N^{F}}\sum_{j}^{N^{narrow}}\left(1-\frac{|R_{D,narrow}-D_{S,k,i,j}|}{D_{S,max}}\right)$$

(24) $$D^{wide}=\sum_{k}^{N^{T}}\sum_{i}^{N^{F}}\sum_{j}^{N^{wide}}\left(1-\frac{|R_{D,wide}-D_{S,k,i,j}|}{D_{S,max}}\right)$$

where $N^{T}=2$ is the number of training trajectories, $N^{F}=4$ is the number of followers, $N^{narrow}$ and $N^{wide}$ are the numbers of simulation steps for narrow and wide formation, $B_{S}$ and $D_{S}$ are bearing and distance to the neighbour measured with sonar, $B_{S,max}=$ 90 deg is the maximum angular range of sonar, $R_{D,narrow}=5$ and $R_{D,wide}=15$ are desirable distances to the neighbour for the narrow and wide formations, and $D_{S,max}=15$ is the maximum range of sonar for detecting other swarm members. The remaining parameters of the simulations are as follows: maximum speed of followers—$V_{max}^{F}=$ 2 m/s, the time interval between successive estimations of neighbour state by the TSBA and decisions of the algorithm—$\Delta t_{E}=0.5$ s, the interval between successive NSCSUV decisions—$\Delta t_{D}=1$ s, the interval between successive sonar measurements—$\Delta t_{E}=2$ s, simulation’s time step—$\Delta t_{E}=0.1$ s.

To avoid unnecessarily prolonging the evaluation of algorithms, simulations were interrupted in three situations. First, if any follower was more than 50 m away from the leader. Second, when all followers made the same decisions regarding the direction of movement and speed for a short period. Third, when the distance between any pair of vehicles was less than 2 m, it was considered a collision. Each of the above situations shortened the simulations, decreased $N^{I}$, and thus also reduced the fitness of an evaluated network.

The training process for each algorithm was run 20 times. Of all 20 instances of algorithms produced as a result of the training process, one representative instance was selected for each of them, characterised by the highest fitness (20). In the case of the NSCSUV, this instance was two neural networks with fixed weights; in the case of the TSBA, it was an algorithm with the following parameters: $T_{\theta }=2.4$ s, $T_{D}^{1}=8.4$ m, $T_{D}^{2}=5.2$ m, $p_{1}=1.5$, $p_{2}=1$, $p_{3}=0.1$, $p_{4}=0.5$.

Both representative instances were validated in MOOS-IvP 24.8 environment. For this purpose, each algorithm was implemented as a separate MOOS-IvP behaviour. The standard uSimMarineV22 application used to model vehicles was replaced by one that implements the neural model specified in Section 2. The behaviours were fed with information from the application implementing the sonar operation. For validation tests, instead of 4 followers in the swarm, a simpler version with 2 followers was used.

To bring the simulation conditions closer to real conditions and to the situation in which the followers are low-cost vehicles with inaccurate underwater navigation, the heading, speed, and, consequently, the vehicles’ positions were deliberately disturbed. For heading, a change in one direction was assumed, mimicking actual compass drift. The maximum heading error was equal to 5 degrees. A similar approach was used for speed, mimicking a situation in which the speedometer either underestimates or erroneously overestimates the speed. In this case, the maximum error amounted to 0.3 m/s.

**Figure 8:**
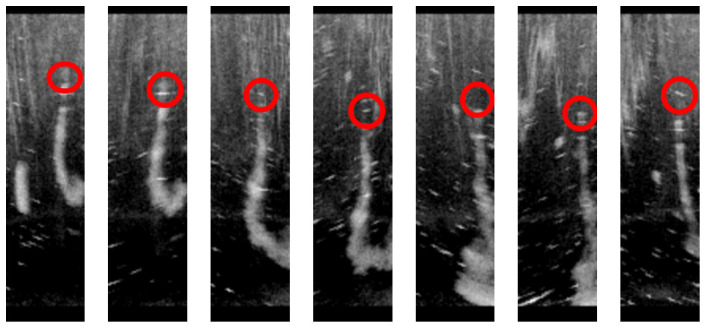
The tracked object (red circle) is selected based on intensity-based detection within a region of interest and proximity to the expected object location.

A similar procedure was used for the sonar, generating the distance to the neighbour and the bearing to it. In this case, the distance was reduced by up to 2 m, corresponding to a situation in which, instead of the echo from the 1.5 m vehicle, the sonar detects the echo from the water stream emitted by the engines behind the vehicle—see Figure 8 in the text below. The bearing error was either positive or negative, with a maximum value of 10 degrees. This corresponds to a situation where the vehicle’s echo is not a point but a diffuse spot shifted to the right or left, with the centre indicating the vehicle’s position.

**Figure 9:**
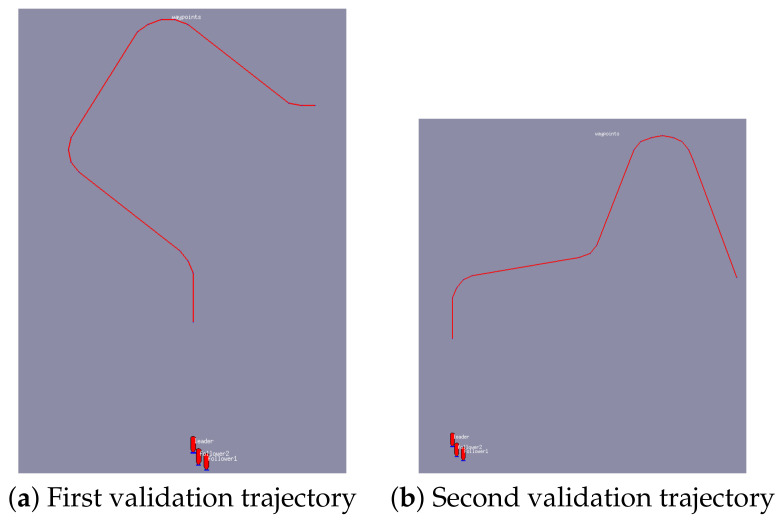
Trajectories used in validation phase.

The algorithms were validated on manually planned trajectories depicted in Figure 9. Two variants of each trajectory were used. In the first variant, the leader moved at a constant speed of 1 m/s, regardless of the formation. In the second variant, the leader moved at 1 m/s on turns and at 0.5 m/s on straight sections. Regardless of the trajectory variant, the swarm moved in a narrow formation on turns and in a wide formation on the straight sections.

To compare the algorithms, four evaluation criteria were used, i.e., $F_{D}$, $F_{B}$, $F_{V}$, and $F_{\theta }$. The first two criteria determine the average distance and bearing error for one iteration. The third and fourth criteria assess the algorithm’s stability with respect to the desired heading and speed. Solutions that rarely change vehicle motion parameters are preferred. All four criteria are defined below:

(25) $$F_{D}=\frac{\sum_{i}^{N^{iter}}F_{D,i}}{N^{iter}}$$

(26) $$F_{D,i}=\left\{\begin{array}{cc} |R_{D,narrow}-D_{S,i}| & \mathrm{narrow}\;\mathrm{formation}\\ |R_{D,wide}-D_{S,i}| & \mathrm{otherwise} \end{array}\right.$$

(27) $$F_{B}=\frac{\sum_{i}^{N^{iter}}F_{B,i}}{N^{iter}}$$

(28) $$F_{B,i}=\left\{\begin{array}{cc} d_{ang}\left(B_{D,narrow},B_{S,i}\right) & \mathrm{narrow}\;\mathrm{formation}\\ d_{ang}\left(B_{D,wide},B_{S,i}\right) & \mathrm{otherwise} \end{array}\right.$$

(29) $$F_{V}=\frac{\sum_{i=1}^{N^{iter}}|V_{D}\left(i\right)-V_{D}\left(i-1\right)|}{N^{iter}}$$

(30) $$F_{\theta }=\frac{\sum_{i=1}^{N^{iter}}d_{ang}\left(\theta_{D}\left(i\right)-\theta_{D}\left(i-1\right)\right)}{N^{iter}}$$

where $N^{iter}$ is the number of iterations needed by the leader to travel from the starting point to the last waypoint on the trajectory. $N^{iter}$ varies depending on the trajectory number and its variant.

**Figure 10:**
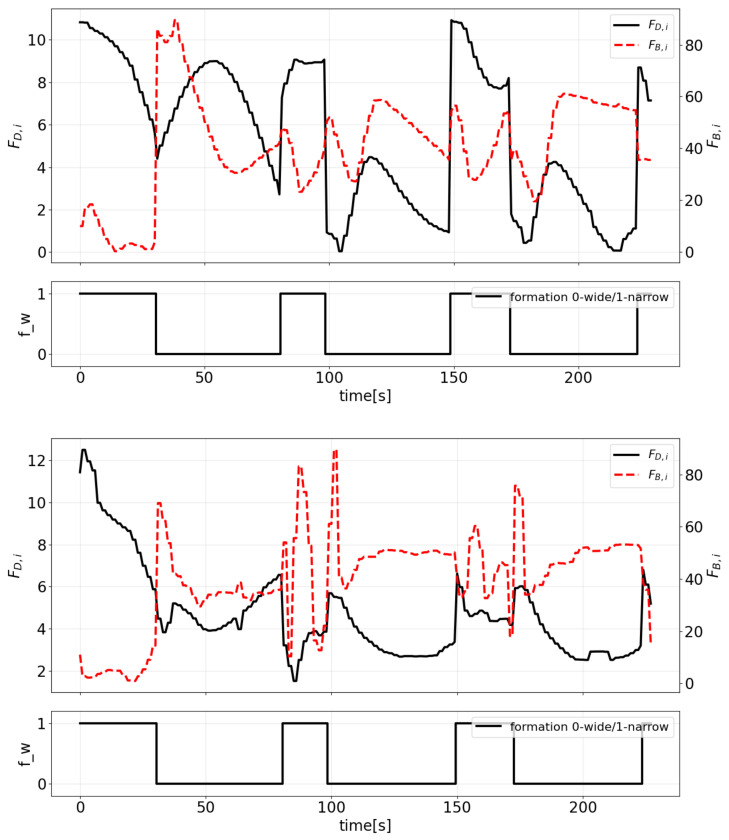
Errors $F_{D}$ and $F_{B}$ for example missions along trajectory depicted in Figure 9: upper figure—NSCSUV, bottom figure—TSBA.

Two representative instances, TSBA and NSCSUV, selected after the training process, were run 15 times for each validation trajectory and each trajectory variant. Each run involved rating two followers—say, follower1 and follower2—according to the criteria defined above. These ratings were then averaged and presented in Table 2, Table 3, Table 4 and Table 5. Errors $F_{D}$ and $F_{B}$ for example missions along the trajectory depicted in Figure 9 are also given in Figure 10.

These tables show that the TSBA is more effective than the NSCSUV with respect to criteria $F_{D}$, $F_{V}$, and $F_{\theta }$. This means that TSBA-controlled followers maintain the desired distance better and also consume less energy through stable heading and speed manoeuvring than their NSCSUV-controlled counterparts. The NSCSUV is more efficient only in terms of $F_{B}$, meaning that NSCSUV-controlled vehicles maintain their desired bearing better than their TSBA-controlled counterparts. However, this comes at the cost of higher energy consumption and frequent course and speed changes.

**Figure 11:**
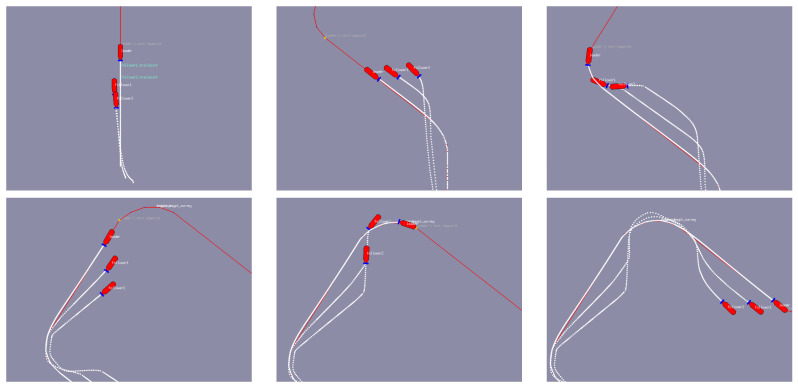
Example of NSCSUV-controlled vehicle behaviour on trajectory no. 1.

**Figure 12:**
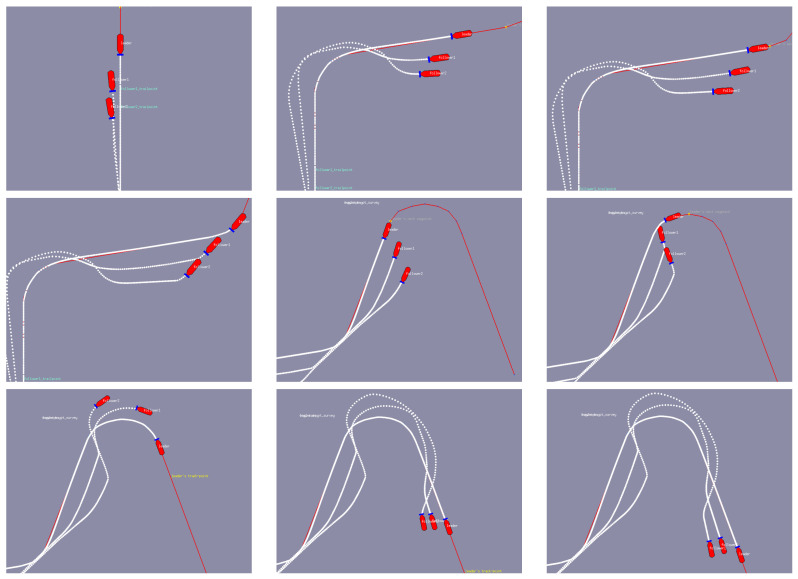
Example of NSCSUV-controlled vehicle behaviour on trajectory no. 2.

**Figure 13:**
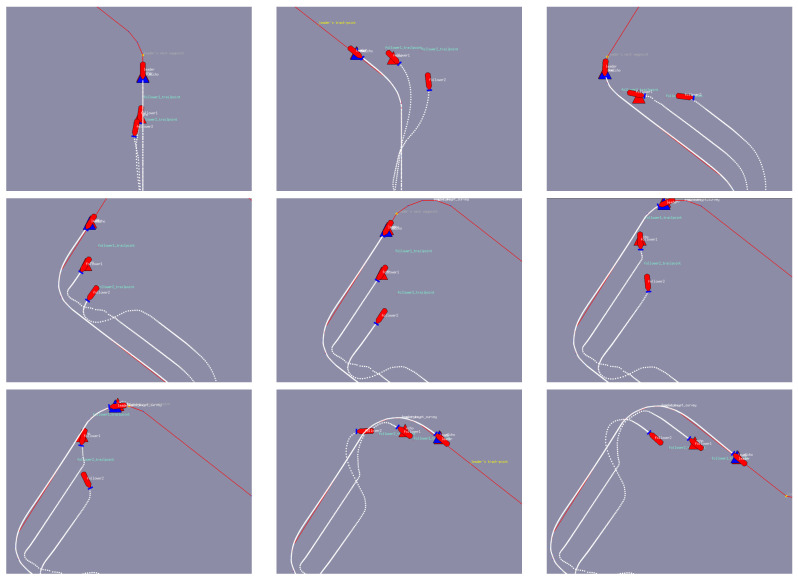
Example of TSBA-controlled vehicle behaviour on trajectory no. 1.

Examples of the behaviour of both algorithms are shown in Figure 11, Figure 12 and Figure 13. They show what was described above, namely that the vehicles controlled by TSBA do not keep up with their tracked neighbour, which is why maintaining the bearing is less accurate in this case. However, they also demonstrate the greater inertia of NSCSUV-controlled vehicles, particularly noticeable on trajectory turns.

**Figure 14:**
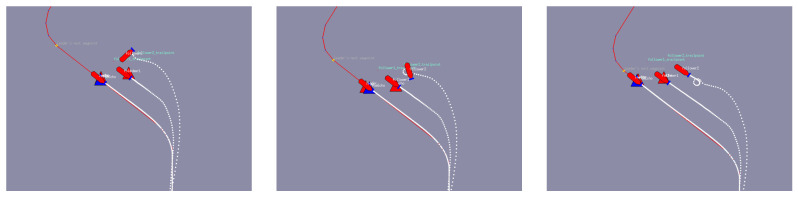
Visualisation of the situation when the follower is late in reducing speed, overtakes the track point, and consequently has to turn back.

The results from the tables also show that slowing the swarm on straight sections of the trajectory, where vehicles must change from narrow to wide formation, positively affects the algorithms’ efficiency. Unfortunately, in specific cases, this procedure can cause the effect shown in Figure 14, when follower2, controlled by the TSBA, first tries to quickly catch up with the swarm by increasing its speed, and when the leader reduces its speed on the straight section, follower2 overtakes the track point and consequently turns back.

To eliminate this effect, the TSBA procedure for determining track point coordinates was slightly modified. Instead of (7) and (8), the following was used:

(31) $$x_{T}\left(t\right)=x_{E}\left(t\right)+R_{D}^{1}cos\left(A_{360}\left(\theta_{E}\left(t\right)+B_{D}\right)\right)$$

(32) $$y_{T}\left(t\right)=y_{E}\left(t\right)+R_{D}^{1}sin\left(A_{360}\left(\theta_{E}\left(t\right)+B_{D}\right)\right)$$

(33) $$R_{D}^{1}=\left\{\begin{array}{cc} R_{D} & d_{xy}\left({\left(x,y\right)}_{A},{\left(x,y\right)}_{E}\right)<R_{D}\\ 0.5R_{D} & \mathrm{otherwise} \end{array}\right.$$

Applying the above procedure prevented the situations presented in Figure 14. Unfortunately, the TSBA results turned out to be worse than those obtained by the original.

One aspect that was not analysed in the comparative tests was the communication overhead, as the comparison focused primarily on the ability of both algorithms to maintain the desired formation. Nevertheless, the communication requirements of the two approaches can be compared based on their operating principles. Both TSBA and NSCSUV require communication only when the leader changes its active waypoint, and therefore their communication frequency is the same and depends on the frequency of waypoint changes. If the complete mission plan is known a priori to all vehicles, both algorithms require only the identifier of the newly activated waypoint to be transmitted. If the mission plan is not known in advance, TSBA requires information about the new waypoint and the tracked object’s intended motion parameters, including the desired speed, depth, and heading, as well as the required formation parameters. In contrast, NSCSUV relies on FLS observations to determine the motion of the tracked vehicle and requires only information defining the desired formation. Thus, although both algorithms have the same communication frequency, TSBA may require a larger payload per communication event when the complete mission plan is not available a priori.

### 4.3. Verification Under Real Conditions

The final TSBA verification was conducted at Lake Kosobudno in northern Poland. The swarm was tested with one follower or, as in the case of validation tests, with two followers. Three different vehicles were used in the tests, i.e., the test vehicle shown in Figure 1 as follower1, and two other vehicles, i.e., the leader and follower2, both of a different design than the test vehicle. Both followers were equipped with exactly the same TSBA instance, i.e., an instance that was optimised during the training process and then validated in simulated conditions.

The field experiments were limited to the validation of the TSBA and did not include the NSCSUV. This decision was primarily motivated by the mismatch between the dynamic properties of the vehicles used in the field tests and the vehicle model employed during NSCSUV training. One of the follower vehicles had a substantially different mechanical configuration, while the second, although originally used to develop the training model, had undergone hardware modifications before the experimental campaign that altered its dynamic properties. Consequently, the trained NSCSUV could not be deployed on either vehicle without prior adaptation, retraining, and validation. Moreover, simulation tests indicated that the NSCSUV responded more slowly to trajectory changes than the TSBA. Given the restricted dimensions of the test area and the presence of a pier, shallow regions, and dense aquatic vegetation, testing an unvalidated controller with a slower response could have posed an unacceptable risk to the experimental vehicles. Therefore, the objective of the field campaign was limited to validating the feasibility and effectiveness of the TSBA under real sensing, communication, and environmental conditions.

All the tests were conducted along a roughly rectangular trajectory, with longer straight sections and curves. The distances between the waypoints, which constituted the vertices of the rectangle along which the leader moved, were approximately 100–200 m. At a leader speed of 1m/s, this means a waypoint change after 100 and 200 s. At a speed of 2 m/s, this change occurred twice as fast, i.e., after 50 and 100 s, respectively. This means that the TSBA was tested under stable conditions that did not require frequent manoeuvres resulting from unstable leader behaviour.

Some tests took place underwater, but in this case, it was difficult to assess the TSBA’s effectiveness because the vehicles were invisible, and determining their precise underwater position was impossible because the followers were equipped with low-cost, imprecise navigation systems. Furthermore, due to the lake’s maximum depth of 6 m, the operation of sonar, which estimates the relative position of the tracked neighbour, was significantly hampered by the large number of reflections originating from the lake bottom or surface.

Finally, to minimise the influence of external factors on the TSBA’s effectiveness, the algorithm was validated on the surface. The relative position of the tracked object, as well as each follower position, was determined using the GPS and then disturbed in a manner similar to that during validation tests under simulation conditions.

During all surface tests, the followers were tasked with tracking the leader in two different formations: a narrow formation and an inverted V formation with the leader at the head. In the narrow formation, the desired distance between vehicles was 10 m. This distance was intentionally increased compared to the simulation tests. This was because each vehicle was equipped with a buoy with a WiFi antenna attached to the rear of the vehicle on a 3 m cable. This buoy was used for continuous communication with the vehicles. This solution was necessary for the safety of the vehicles, which are solely research platforms.

Formation V required vehicles to maintain a distance of 15 m from the leader and a bearing on it, depending on the follower number. For follower1, the bearing was 315 degrees and for follower2, it was 45 degrees.

**Figure 15:**
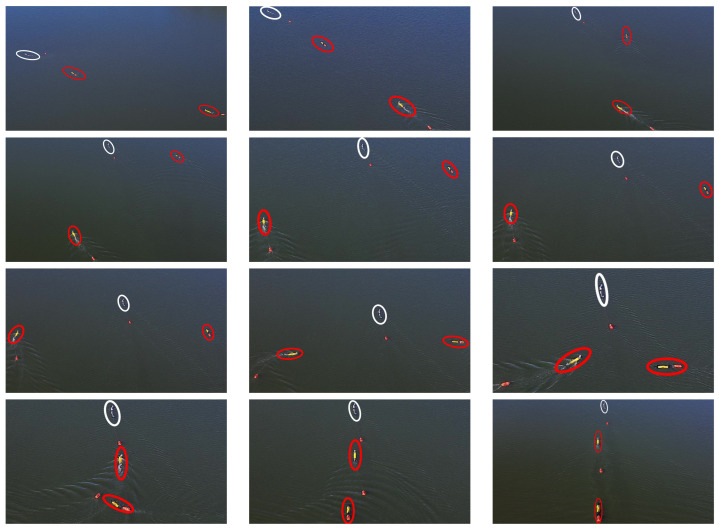
The first part of the swarm’s mission on Lake Kosobudno: leader—white ellipse, followers—red ellipses.

**Figure 16:**
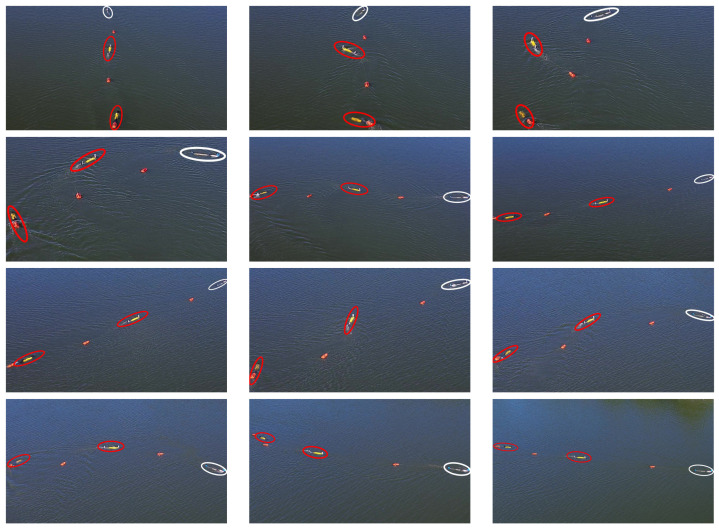
The second part of the swarm’s mission on Lake Kosobudno: leader—white ellipse, followers—red ellipses.

An example of a vehicle mission on Lake Kosobudno is shown in Figure 15 and Figure 16. Figure 15 shows the first part of the mission when the vehicles start in a narrow formation, then change to a V formation, which, after travelling 50 m, changes back to a narrow formation. Figure 16 shows the next part of the mission when the vehicles first turn right, travel 50 m straight in a narrow formation, turn right again, and finish after another 50 m along a straight section of the trajectory, also in a narrow formation.

Because the photographs shown in both figures were taken from a flying drone at a certain height, the vehicles featured in them were, in some cases, barely visible. To facilitate the locating of vehicles and to determine their current orientation (heading), each vehicle is surrounded by an ellipse: the leader by a white ellipse, and the followers by a red ellipse.

**Figure 17:**
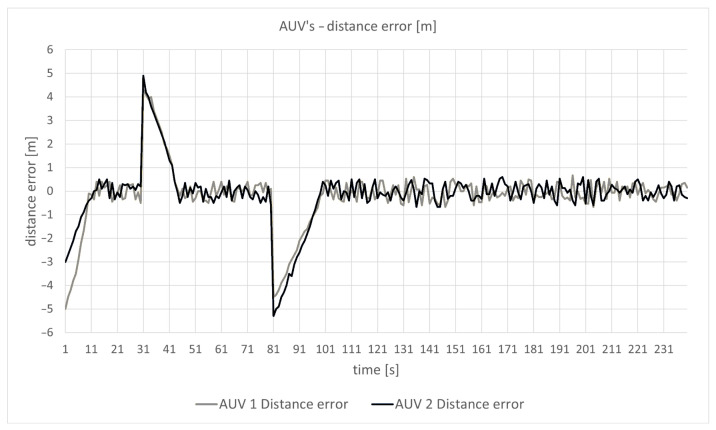
Distance error for follower1 and follower2 (AUV1 and AUV2).

**Figure 18:**
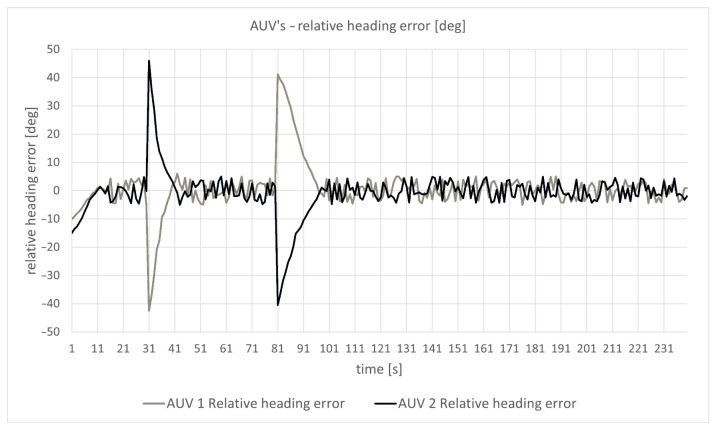
Relative heading error for follower1 and follower2 (AUV1 and AUV2).

The results of a similar mission are also presented in Figure 17 and Figure 18. In this case, the distance and heading errors for both followers are presented.

Three evaluation metrics were employed to comprehensively assess the performance and quality of the TSBA algorithm under real-world operating conditions. The first metric was the average time required to achieve the target formation position across all validation missions, which served as a measure of the algorithm’s responsiveness and convergence efficiency. For each mission, this time was defined as the interval between issuing the command to change formation and the moment the vehicle successfully reached the desired relative position within the new formation. The final metric value was obtained by averaging the transition times over all missions. Shorter average transition times were interpreted as indicative of superior algorithm performance, as they reflected the vehicle’s ability to rapidly determine and execute an appropriate course and velocity profile to assume the commanded position.

The second evaluation metric was the MSE—Mean Squared Error—of the distance between AUVs in the target formation, used to quantify the precision and stability of maintaining the prescribed inter-vehicle separation once the formation had been established. Lower values of this metric indicated reduced fluctuations around the desired spacing and, consequently, more accurate formation keeping.

The third metric considered was the MSE of the relative heading in the target formation, which characterised the accuracy and consistency with which the desired relative orientation between vehicles was maintained. Similar to the distance-related metric, smaller relative heading deviations were associated with improved control quality and greater robustness of the algorithm.

Both MSE metrics were calculated based on the differences between the desired (target) and measured values of distance and relative heading, respectively. Importantly, these calculations were performed exclusively over trajectory segments in which the vehicle had already reached and was maintaining the target formation position. Transitional segments corresponding to active formation changes were intentionally excluded from the analysis in order to isolate steady-state performance from transient manoeuvring effects and to provide a more representative assessment of formation maintenance quality.

The average values of these three performance metrics during the experimental validation of the TSBA algorithm under real-world conditions are summarised in Table 6. The reported mean values were calculated over ten independent missions conducted along similar trajectories, providing a representative assessment of the algorithm’s performance under repeatable operating conditions. These results provide a quantitative basis for evaluating the effectiveness, precision, and dynamic response characteristics of the proposed control approach. Additionally, the standard deviation values are also presented, illustrating the variability and dispersion of the measured results around their mean values across the ten missions. Consequently, they provide further insight into the repeatability, consistency, and robustness of the proposed control approach under real-world operating conditions.

Overall, all tests demonstrated the effectiveness of the TSBA in controlling swarms in various formations, even under conditions of imprecise navigation and inaccurate information about the tracked object. For more severe errors in the tracked object’s location, the only effect observed was more frequent and larger heading corrections by the followers. However, these corrections were not sufficient to disrupt the swarm’s formation. Nevertheless, they resulted in higher energy consumption.

Another negative phenomenon observed was one we had previously encountered during validation testing and is shown in Figure 14. On curves or immediately after curves, vehicles failed to brake in time and were too far ahead, resulting in vehicle u-turns and, in some cases, collisions. To avoid such situations, a modification of the TSBA was used, previously tested during validation, and based on functions (31) and (32). The accuracy of maintaining formation was no longer as satisfactory as with the original TSBA (formation assessment was performed solely visually), but this eliminated the risk of collision.

## 5. Conclusions

This paper presents and compares two different swarm control algorithms, i.e., the TSBA, which represents a simpler algorithmic approach, and the NSCSUV, which is implemented as a neural network. Both algorithms are based on the same assumption: each vehicle in the swarm has only one neighbour, which it tracks. Even if other detected objects appear near the vehicle, such as obstacles or other vehicles in the swarm that have deviated from their intended trajectory, this assumption makes it easier to distinguish these objects from the one that should be tracked.

The algorithms were compared in simulation settings; however, to enable subsequent verification of one of them in real-world conditions using an actual underwater vehicle, the comparative simulation tests were preceded by the construction of a reliable model of the vehicle. As it turned out, the classic model-driven approach completely failed, requiring the use of a data-driven approach and neural networks, which handled the problem perfectly. The key in this case was model modularisation and separate modelling of speed and heading based on data from the vehicle’s drives. Another crucial issue was the separate modelling of motion on a straight line and around curves.

The comparison of algorithms was also preceded by their optimisation using an evolutionary approach and a previously constructed neural vehicle model.

The comparison of the algorithms demonstrated the effectiveness of each in controlling a swarm in two different formations. As it turned out, the TSBA was more effective in maintaining the desired distance to the tracked neighbour, while the NSCSUV was more accurate in maintaining the desired bearing. However, a significant advantage of the TSBA over its competitor is its greater vehicle control stability in the form of smaller and smoother changes in the vehicle’s heading and speed, which translates into lower energy consumption.

Tests of the TSBA under real-world conditions confirmed the algorithm’s effectiveness in controlling swarms in various formations, not just those tested in simulations. Some issues with vehicle speeds not properly matching the conditions, which resulted in undesirable vehicle behaviour and sometimes even collisions, were resolved. However, the implemented solution slightly degraded the TSBA performance in maintaining the desired formation.

An important limitation of the TSBA is its dependence on a priori knowledge of the mission parameters of the tracked vehicle. This dependence does not restrict the algorithm to missions with strictly stable trajectories, provided that changes in the mission parameters can be communicated to the swarm at a rate sufficient to maintain an up-to-date representation of the intended motion of the tracked object. A degradation of formation-keeping performance can be expected only when the mission parameters change so frequently that the corresponding updates cannot be transmitted through the acoustic communication channel in a timely manner. Under such conditions, a follower may operate with outdated information about the intended speed, depth, heading, or formation parameters of the object it tracks. The FLS then becomes the primary source of information about the tracked object. However, in the considered system, FLS measurements are available every 2 s and provide only uncertain relative position measurements, without direct information about the tracked object’s speed, heading, or depth. Consequently, rapid changes in mission parameters that cannot be adequately reflected in the communicated mission data may increase motion-estimation errors and degrade formation-keeping performance. Quantifying the relationship between the rate of mission-parameter changes, communication constraints, and the resulting performance degradation requires a dedicated sensitivity analysis and constitutes an important direction for future work.

Generally, tests of both algorithms, under both simulation and real-world conditions, have shown that controlling a swarm of underwater vehicles in simple formations does not require very complex algorithms. Feeding the algorithms with reliable information about the location of the tracked object seems to be a much more difficult problem than the control itself. This was confirmed by tests on a shallow lake using sonar, which had significant difficulties in accurately tracking the object. This was due to both very noisy sonar scans containing reflections from the bottom and water surface, as well as visible water streams generated by the vehicle’s thrusters.

## Figures and Tables

**Table 1:** Results of speed and heading models.

Model	$E_{max}$	$E_{mean}$	σ
SM	1.068 [m/s]	0.107 [m/s]	0.113 [m/s]
HM	66.06 [°]	14.25 [°]	17.14 [°]

**Table 2:** Results for variant no. 1 (1 m/s along the whole trajectory) of trajectory no. 1 (Figure 9a).

		$F_{D}$ [m]	$F_{B}$ [deg]	$F_{V}$ [m/s]	$F_{\theta }$ [deg]
TSBA	Mean follower1	5.65	59.29	0.07	5.29
	Mean follower2	7.20	55.99	0.07	6.20
	Max follower1	5.92	61.64	0.07	5.75
	Max follower2	7.82	64.29	0.07	6.84
NSCSUV	Mean follower1	9.53	38.21	0.18	10.43
	Mean follower2	9.72	50.28	0.15	8.08
	Max follower1	9.62	44.25	0.21	11.26
	Max follower2	10.05	55.29	0.21	10.43

**Table 3:** Results for variant no. 1 (1 m/s along the whole trajectory) of trajectory no. 2 (Figure 9b).

		$F_{D}$ [m]	$F_{B}$ [deg]	$F_{V}$ [m/s]	$F_{\theta }$ [deg]
TSBA	Mean follower1	6.23	61.14	0.05	5.19
	Mean follower2	7.32	63.95	0.09	6.04
	Max follower1	6.78	63.55	0.07	5.62
	Max follower2	8.42	67.95	0.10	7.25
NSCSUV	Mean follower1	8.99	39.33	0.20	12.51
	Mean follower2	12.75	46.59	0.17	11.96
	Max follower1	10.87	42.96	0.22	13.24
	Max follower2	14.35	49.66	0.21	13.73

**Table 4:** Results for variant no. 2 (1 m/s on turns and 0.5 m/s on straight sections) of trajectory no. 1 (Figure 9a).

		$F_{D}$ [m]	$F_{B}$ [deg]	$F_{V}$ [m/s]	$F_{\theta }$ [deg]
TSBA	Mean follower1	5.86	45.32	0.02	2.19
	Mean follower2	6.65	47.14	0.03	3.13
	Max follower1	6.26	46.23	0.03	2.66
	Max follower2	7.22	49.67	0.03	3.63
NSCSUV	Mean follower1	3.44	30.37	0.08	12.08
	Mean follower2	3.92	41.03	0.08	10.32
	Max follower1	3.98	35.70	0.08	13.20
	Max follower2	4.36	45.15	0.08	13.38

**Table 5:** Results for variant no. 2 (1 m/s on turns and 0.5 m/s on straight sections) of trajectory no. 2 (Figure 9b).

		$F_{D}$ [m]	$F_{B}$ [deg]	$F_{V}$ [m/s]	$F_{\theta }$ [deg]
TSBA	Mean follower1	5.48	43.95	0.02	2.05
	Mean follower2	6.65	47.81	0.05	2.87
	Max follower1	6.07	45.88	0.04	2.46
	Max follower2	7.71	50.21	0.07	3.31
NSCSUV	Mean follower1	6.12	33.04	0.11	10.11
	Mean follower2	5.02	38.72	0.10	11.76
	Max follower1	6.77	35.88	0.11	10.94
	Max follower2	6.21	40.22	0.11	13.23

**Table 6:** Results of field tests, ${\bar{t}}_{W}$, ${\bar{t}}_{N}$ are the average times required to achieve the wide and narrow formation positions across all validation missions for comparison purposes with the tests under simulation conditions. The table also includes evaluation criteria used in these tests, i.e., $F_{D}$—Equation (25) and $F_{B}$—Equation (27).

AUV	${\mathrm{MSE}}_{\mathrm{dist}.}$ [m]	$\sigma_{\mathrm{dist}.}$ [m]	$F_{D}$ [m]	${\mathrm{MSE}}_{\mathrm{rel}\_\mathrm{head}.}$ [deg]	$\sigma_{\mathrm{rel}\_\mathrm{head}.}$ [deg]	$F_{B}$ [m]	${\bar{t}}_{W}$ [s]	${\bar{t}}_{N}$ [s]
Follower 1	0.10	0.32	0.27	8.08	2.84	2.46	14	18
Follower 2	0.09	0.30	0.27	7.38	2.72	2.36	13	17

## Data Availability

The data that support the findings of this study are available from the corresponding author, T.P., upon reasonable request.
